# The Novel-Natural-Killer-Cell-Related Gene Signature Predicts the Prognosis and Immune Status of Patients with Hepatocellular Carcinoma

**DOI:** 10.3390/ijms24119587

**Published:** 2023-05-31

**Authors:** Minjun Li, Juntao Huang, Guohua Zhan, Yuankuan Li, Chunye Fang, Bangde Xiang

**Affiliations:** 1 Department of Hepatobiliary Surgery, Guangxi Medical University Cancer Hospital, Nanning 530021, China; liminjunm6142@163.com (M.L.); huangjuntao@sr.gxmu.edu.cn (J.H.); zhanguohuadoctor@163.com (G.Z.); yuankuanli@163.com (Y.L.); 18276149659@163.com (C.F.); 2 Key Laboratory of Early Prevention and Treatment for Regional High Frequency Tumor, Ministry of Education, Nanning 530021, China

**Keywords:** hepatocellular carcinoma, natural killer cell, prognosis

## Abstract

The current understanding of the prognostic significance of natural killer (NK) cells and their tumor microenvironment (TME) in hepatocellular carcinoma (HCC) is limited. Thus, we screened for NK-cell-related genes by single-cell transcriptome data analysis and developed an NK-cell-related gene signature (NKRGS) using multi-regression analyses. Patients in the Cancer Genome Atlas cohort were stratified into high- and low-risk groups according to their median NKRGS risk scores. Overall survival between the risk groups was estimated using the Kaplan–Meier method, and a NKRGS-based nomogram was constructed. Immune infiltration profiles were compared between the risk groups. The NKRGS risk model suggests significantly worse prognoses in patients with high NKRGS risk (*p* < 0.05). The NKRGS-based nomogram showed good prognostic performance. The immune infiltration analysis revealed that the high-NKRGS-risk patients had significantly lower immune cell infiltration levels (*p* < 0.05) and were more likely to be in an immunosuppressive state. The enrichment analysis revealed that immune-related and tumor metabolism pathways highly correlated with the prognostic gene signature. In this study, a novel NKRGS was developed to stratify the prognosis of HCC patients. An immunosuppressive TME coincided with the high NKRGS risk among the HCC patients. The higher KLRB1 and DUSP10 expression levels correlated with the patients’ favorable survival.

## 1. Introduction

Hepatocellular carcinoma (HCC) is among the most prevalent malignancies worldwide [1], and patients with early-stage HCC are eligible to receive curative treatments [2]; however, over 50% of patients experience tumor relapse within 5 years of curative resection, with poor long-term overall survival (OS) [3,4]. The efficacy of systemic therapies, such as sorafenib, for patients with advanced-stage HCC remained low [5] until immune checkpoint inhibitors (ICIs) were recommended [6]. Unfortunately, the objective response rates of ICI therapies or the combination of ICI and targeted therapies only ranged from 20% to 30% [7,8]. One important reason for the unsatisfactory response to systemic therapies is that HCC involves a highly complex and heterogeneous tumor microenvironment (TME).

The development and progression of malignancies occur in a continuously evolving TME with an altered immune landscape [9]. Exploring the relationships between malignant cells, lymphocytes, and other intratumoral components in the TME is vital in anticancer treatments. Single-cell RNA sequencing (scRNA-seq) technology provides more opportunities to analyze the TME, and an increasing number of studies based on scRNA-seq have enriched the understanding of the TME. These studies have focused primarily on the prognostic value of T cells or macrophages and revealed the non-cytotoxic state of tumor-infiltrating T cells or the immunosuppressive role of macrophages in the immune landscape [10,11,12]; however, only few studies have considered the essential role of natural killer (NK) cells in the immune system. NK cell infiltration positively correlated with a favorable prognosis in HCC [13,14]. Previous reports have suggested that NK cells are vital immunoregulative components in anti-cancer immunity by regulating T-cell function [15,16]. In addition, HCC patients with a higher level of intratumoral NK cell infiltration may respond better to sorafenib treatment [17]. These findings collectively indicate that dysregulation of NK cell function contributes to HCC progression [18]. Given the prognostic value of NK cells in tumor immune regulation, it is crucial to investigate the immune microenvironment of patients with different NK cell gene expression levels and to evaluate their corresponding survival significance.

In this work, we integrated single-cell transcriptome and bulk RNA sequencing to ex the expression profiles of NK cell marker genes to develop an NK-cell-related gene signature (NKRGS) to stratify the prognosis of HCC patients and to evaluate the immune infiltration status in patients with diverse NKRGS risks.

## 2. Results

### 2.1. ScRNA-seq Analysis Identified NK-Cell-Related Genes

**Figure 1:**
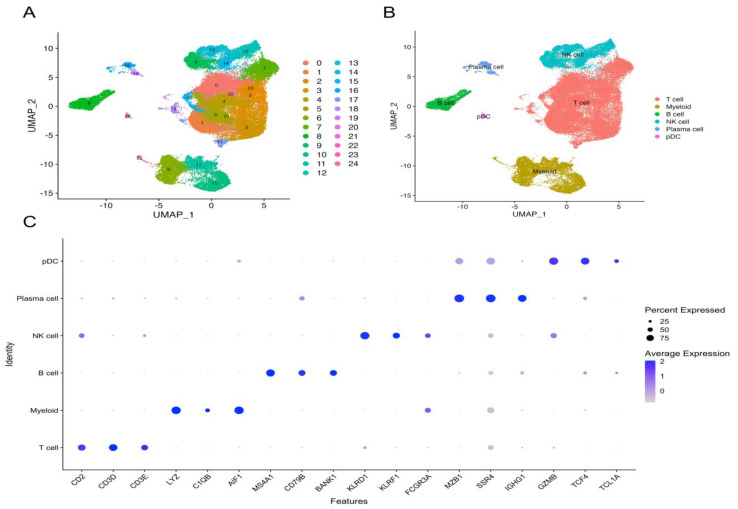
Identification of NK cells using scRNA–seq analysis. (**A**) Twenty–five clusters were identified using the UMAP algorithm. (**B**) Six cell types were defined using immune cell marker genes. (**C**) The dot plot shows the relative expression levels of the selected marker genes in each defined cell type. The size and color depth of the dots represent the marker gene expression proportion and intensity of each cell type, respectively.

In total, 25 cell clusters were identified (Figure 1A), comprising 60,592 CD45^+^ immune cells, and 25 cell clusters were evaluated on the basis of the marker gene expression panel to identify their immune cell type (Figure S1). These clusters were categorized into six cell types according to immune cell marker gene expression levels (Figure 1B), including four NK cell clusters (clusters 9, 12, 13, and 14), one B-cell cluster (cluster 8), two plasma B-cell clusters (clusters 16 and 19), one plasma dendritic cell (pDC) cluster (cluster 22), four myeloid cell clusters (clusters 6, 10, 11, and 23), and 13 T-cell clusters (clusters 0–5, 7, 15, 17, 18, 20, 21, and 24). The dot plot shows the marker gene expression levels of the six identified cell types (Figure 1C). NK-cell-related genes in four NK cell clusters were screened using the selection criteria. Finally, we identified 111 NK-cell-related marker genes by intersecting each NK cell cluster.

### 2.2. NKRGS Risk Model Predicted the Prognoses of Patients with HCC

**Figure 2:**
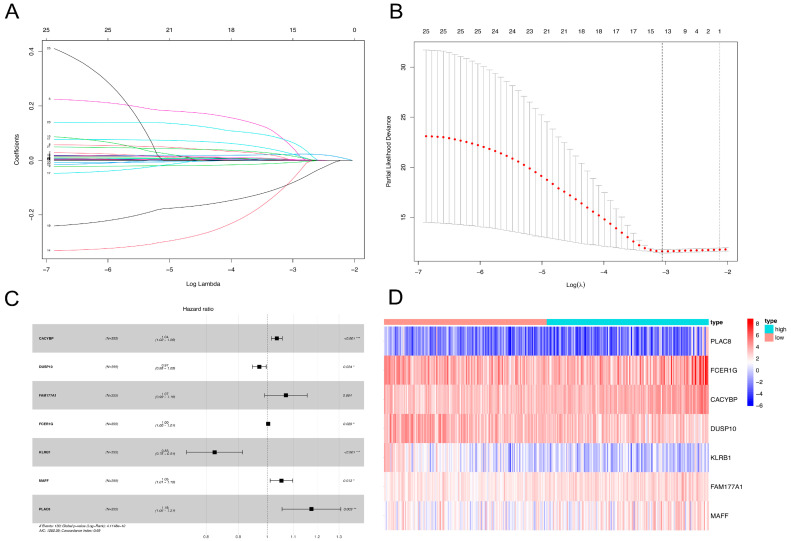
Development of the NKRGS risk model. (**A**,**B**) Least absolute shrinkage and selection operator-regression-selected candidate NK cell marker genes with optimal λ values to construct the NKRGS. (**C**) Forest plot showing the prognostic significance of the selected NK-cell-related genes. (**D**) The heatmap shows the differences in gene expression levels between the NKRGS risk groups.

A univariate Cox analysis was performed on the identified 111 NK-cell-related genes using The Cancer Genome Atlas (TCGA) RNA-seq data, and 25 prognostic-related genes were determined. The least absolute shrinkage and selection operator (LASSO) and multivariate Cox regression analyses further identified nine genes using the optimal *λ* value (Figure 2A,B). Finally, seven NK-cell-related genes were identified, and the NKRGS was constructed, including calcyclin-binding protein (CACYBP), dual specificity phosphatase 10 (DUSP10), family with sequence similarity 177 member A1 (FAM177A1), Fc fragment of IgE receptor Ig (FCER1G), killer cell lectin-like receptor B1 (KLRB1), MAF bZIP transcription factor F (MAFF), and placenta-associated 8 (PLAC8). The prognostic significance of the seven selected NK cell marker genes is shown in Figure 2C. The NKRGS model was developed using the following formula:

risk score = 0.0351 × Exp(CACYBP) − 0.0289 × Exp(DUSP10) + 0.0684 × Exp(FAM177A1) + 0.0034 × Exp(FCER1G) − 0.1920 × Exp(KLRB1) + 0.0521 × Exp(MAFF) + 0.1671 × Exp(PLAC8)(1)

The patients in TCGA cohort were allocated to either a low- (*n* = 178) or high-risk group (*n* = 177) according to their median NKRGS risk scores. The expression heatmap of the seven NKRGS marker genes demonstrates diverse expression levels between the NKRGS risk groups (Figure 2D). Equation (1) was also applied to estimate the risk scores of the patients in the GSE14520 and internal cohorts. The GSE14520 and internal cohorts were allocated to either a low-risk group (*n* = 140 and *n* = 84, respectively) or a high-risk group (*n* = 102 and *n* = 32, respectively).

**Figure 3:**
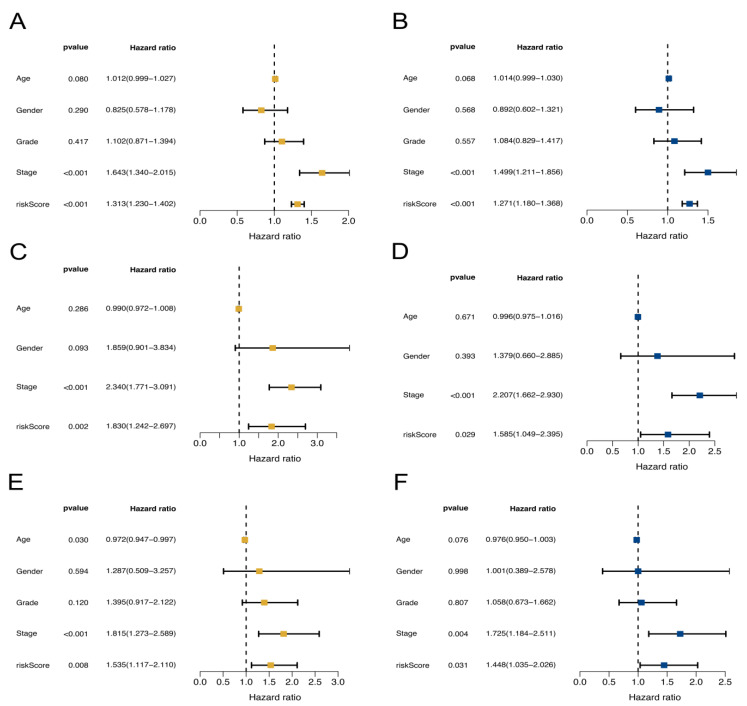
Univariate (**left** panel) and multivariate (**right** panel) Cox regression of the NKRGS risk model. (**A**,**B**) TCGA cohort. (**C**,**D**) GSE14520 cohort. (**E**,**F**) Internal cohort.

**Figure 4:**
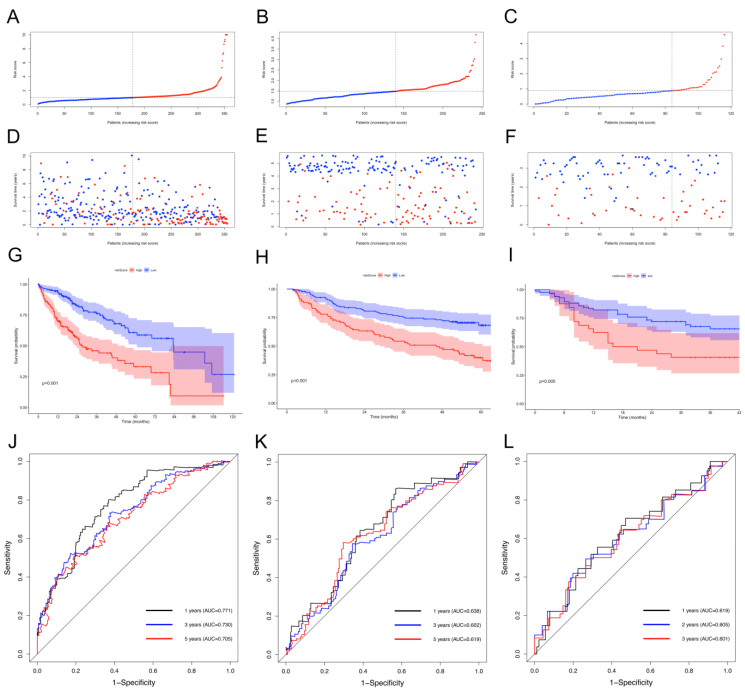
Validation of the NKRGS using TCGA, the GSE14520, and the internal cohorts. (**A**–**C**) Distribution plots showing the proportion of the HCC patients with low and high NKRGS risk scores in TCGA, the GSE14520, and the internal cohorts, respectively. (**D**–**F**) Scatter plots showing increased mortality risk as the risk score increased in TCGA, the GSE14520, and the internal cohorts, respectively. (**G**–**I**) Kaplan–Meier curves of the patients with low and high NKRGS risk scores in TCGA, the GSE14520, and the internal cohorts, respectively. (**J**–**L**) Time-dependent ROC curves showing the prognostic performance of the NKRGS for predicting OS in TCGA, the GSE14520, and the internal cohorts, respectively.

The univariate and multivariate Cox analysis results suggest that both the NKRGS risk score and tumor stage were independent risk factors of the patients in TCGA, internal, and the GSE14520 cohorts (all *p* < 0.05; Figure 3). The distribution plots show increasing risk scores among the patients with HCC in the three cohorts (Figure 4A–C), where the patients with increased risk scores experienced higher risks of mortality (Figure 4D–F). A survival analysis was performed on the three cohorts and revealed that the patients in the low-NKRGS-risk group experienced significantly better OS than their counterparts with high NKRGS risks (all *p* < 0.05; Figure 4G–I). A time-dependent ROC analysis further validated that the NKRGS showed a relatively high predictive ability in TCGA cohort and the two validation cohorts (Figure 4J–L). The area under the curve (AUC) values were 0.771, 0.730, and 0.705 for the 1-, 3-, and 5-year receiver operating characteristic (ROC) curves in TCGA cohort, respectively.

### 2.3. Development and Validation of the NKRGS-Based Nomogram

**Figure 5:**
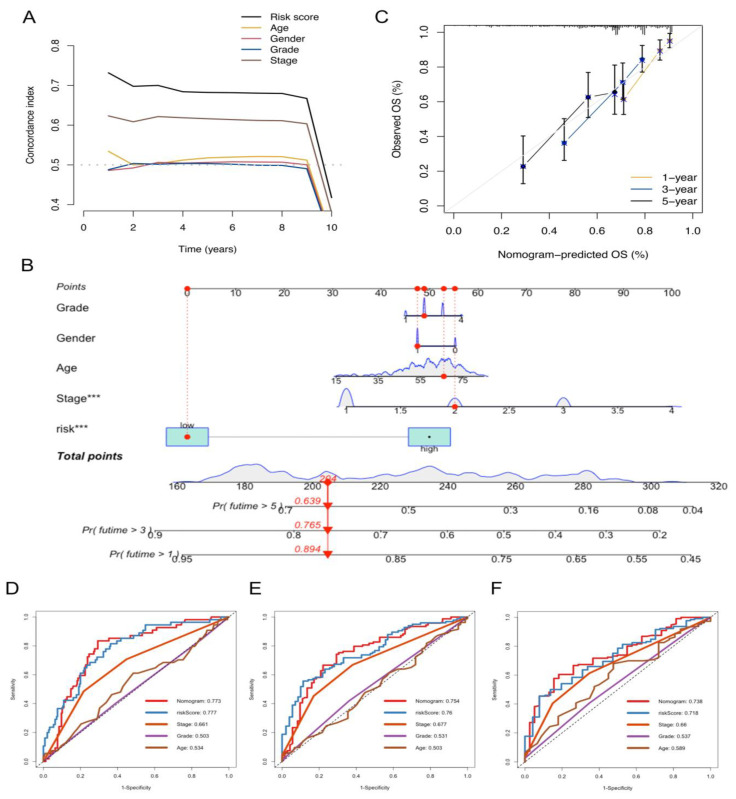
Construction of the NKRGS-based nomogram. (**A**) The NKRGS risk score outperformed the other clinicopathological factors according to the concordance index curve analysis. (**B**) NKRGS-based nomogram for predicting OS in TCGA cohort, with each total point corresponding to the underlying 1-, 3-, and 5-year survival possibilities. (**C**) Calibration curves of the NKRGS-based nomogram for assessing OS. (**D**–**F**) Time-dependent ROC analysis of the nomogram and other variables for predicting 1-, 3-, and 5-year OS, respectively. *** represents *p* values < 0.001.

The prognostic value of the NKRGS was compared with the baseline variables, including disease stage, age, and histological grade. We found that the risk model had the highest concordance index among all the included variables, representing its highest prognostic value (Figure 5A). As the NKRGS risk model was validated to be associated with OS, we developed a NKRGS-based nomogram, combined with other clinicopathological variables, in TCGA cohort (Figure 5B). A relatively strong agreement between the estimated OS and actual values was observed in the calibration curve analysis (Figure 5C), and we evaluated the predictive power of the nomogram compared with those of other clinical characteristics. The AUC values were 0.773, 0.754, and 0.738 for the 1-, 3-, and 5-year ROC curves of the nomogram, respectively. The ROC analysis revealed that the nomogram demonstrated a higher predictive power than the other clinicopathological variables in predicting 5-year OS (Figure 5F) and demonstrated a noninferior performance to the NKRGS risk score in predicting 1- and 3-year OS (Figure 5D,E).

### 2.4. Comparison of Immune Infiltration Profiles between the NKRGS Risk Groups

**Figure 6:**
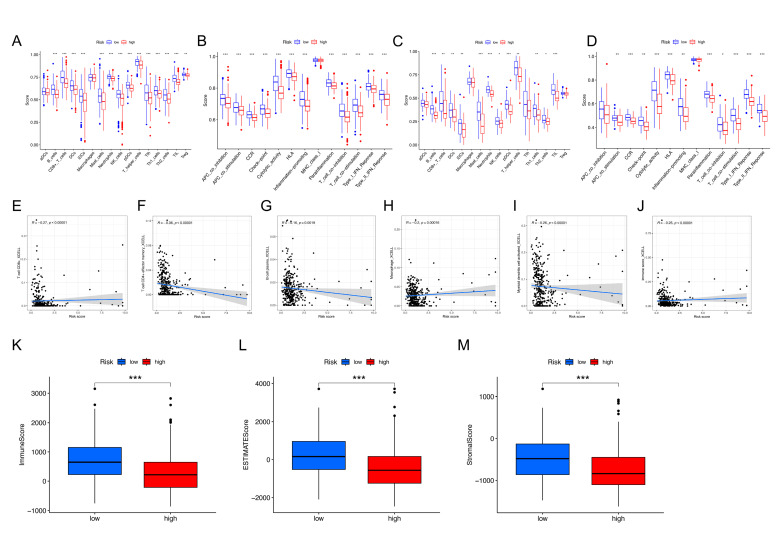
Immune cell infiltration analysis. The enrichment scores of the immune cell types are higher in TCGA cohort (**A**,**B**) and the internal cohort (**C**,**D**). (**E**–**J**) Negative correlations between the NKRGS and immune cell infiltration. (**K**–**M**) ESTIMATE analysis between the NKRGS risk groups. * represents *p* values < 0.05, ** represents *p* values < 0.01, *** represents *p* values < 0.001.

The single-sample gene set enrichment analysis (ssGSEA) revealed that the high-NKRGS-risk patients had significantly lower enrichment scores for major cell types for immune response than those with a low NKRGS risk, which suggests that the high-NKRGS-risk patients were predominantly in an immunosuppressive state (Figure 6A,B), and the internal cohort also validated the same immunosuppressive status (Figure 6C,D). Immune cell correlation analysis also revealed that higher NKRGS risk was negatively associated with immune infiltration, including T cells, B cells, macrophages, and dendritic cells (all *p* < 0.05; Figure 6E–I), as well as a negatively related immune score (*p* < 0.05, Figure 6J). Furthermore, the “estimation of stromal and immune cells in malignant tumor tissues using expression data” (ESTIMATE) analysis [19] revealed that the high-NKRGS-risk patients had significantly lower immune, stromal, and ESTIMATE scores (all *p* < 0.05; Figure 6K–M). These findings collectively demonstrate that the high-NKRGS-risk patients experienced a significantly lower level of immune cell infiltration and a more suppressive tumor immune microenvironment.

### 2.5. NKRGS-Related Differentially Expressed Genes Correlated with Tumor Immunity and Metabolism

**Figure 7:**
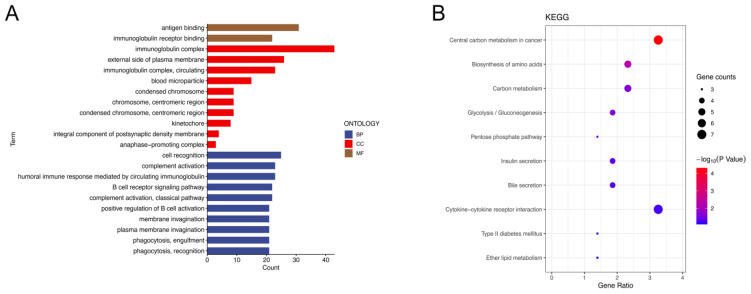
Functional enrichment analysis of the differentially expressed genes. (**A**) GO analysis of NKRGS−related DEGs. BP: Biological process; CC: cellular component; MF: metabolic function. (**B**) KEGG analysis of NKRGS−related DEGs.

In total, 221 differentially expressed genes (DEGs) were identified using the defined gene filter thresholds. We then used these DEGs to perform functional enrichment analyses to investigate the related functional pathways between the NKRGS risk groups. The biological processes in the gene ontology (GO) analysis enriched the cell recognition, complement activation, and positive regulation of B-cell activation pathways. Furthermore, the immunoglobulin complex, external side of the plasma membrane, and circulating immunoglobulin complex were extensively enriched in cellular components. In addition, the metabolic functions enriched the antigen-binding and immunoglobulin-receptor-binding pathways (Figure 7A). The GO analysis revealed that the NKRGS is closely related to immune response. The Kyoto Encyclopedia of Genes and Genomes (KEGG) analysis mainly enriched the metabolic-associated pathways, including the central carbon metabolism in cancer, carbon metabolism, and biosynthesis of amino acids, suggesting that the NKRGS closely correlates with cancer metabolism (Figure 7B).

## 3. Discussion

In recent years, the utility of ICI therapies for patients with HCC has shown an upward trend [20]; however, effectively identifying patients with HCC who will potentially benefit from ICI treatment remains an unsolved problem for clinicians, as a series of randomized controlled trials have failed to prolong the OS of nonviral HCC patients [21]. The highly complicated TME in HCC necessitates the evaluation of the anticancer ability of not only T cells but also other important immunological components. NK cells are important lymphocytes in the anticancer immune response [15,18], and previous reports have indicated that they could regulate the T-cell response and thus affect the response to immunotherapies [15,16,22]. Hence, it is necessary to investigate the potential NK cell expression characteristics in the TME and their relationships with HCC prognosis.

Unlike the other prognostic models proposed for HCC, the novel NKRGS risk model especially focuses on the predictive ability of NK cell-related genes in survival stratification. In this study, we developed a prognostic-related NKRGS, which includes CACYBP, DUSP10, FAM177A1, FCER1G, KLRB1, MAFF, and PLAC8. Within the prognostic gene model, CACYBP, FAM177A1, FCER1G, MAFF, and PLAC8 correlated with unfavorable survival outcomes, whereas DUSP10 and KLRB1 were protective factors for OS in the patients with HCC. The upregulation of CACYBP has been reported to promote tumor progression and lead to significantly worse OS in various cancers [23,24]. Meanwhile, DUSP10 showed higher intratumoral than non-tumoral expression levels in patients with cancer [25]. The downregulation of DUSP10 expression was deemed a tumor suppressor and correlated with tumor migration and distant metastasis [26,27]. FCER1G is related to immune status and contributes to the unfavorable prognosis of patients [28,29,30], and its high expression levels in tumors were positively associated with T-cell dysfunction [28,31]. Furthermore, KLRB1 expression was associated with immune cell infiltration and favorable survival in a pancancer analysis, as higher KLRB1 expression levels positively correlated with CD8^+^ T and γδ T cell infiltration and regulate the immune cell response via interactions with lymphoid and malignant cells [32,33]. This finding is in accordance with our results that a lower NKRGS risk coincides with a significantly higher level of immune cell infiltration, including CD8^+^ T cells. MAFF is a hypoxia-induced gene that promotes the metastatic and self-regenerative capacity of cancer cells [34,35], while PLAC8 contributes to the proliferative and invasive characteristics of malignancies via Wnt/β-Catenin signaling or cell-cycle regulatory pathways [36,37]; however, the prognostic significance of FAM177A1 in HCC remains to be elucidated in subsequent studies.

The established NKRGS risk model exhibited a high prognostic value in TCGA and the validation cohorts, where patients with a high NKRGS risk experienced worse OS. In addition, the constructed NKRGS-based nomogram showed high predictive performance, as the ROC analysis results further demonstrated that the nomogram outperformed the other clinicopathological variables in estimating patients’ prognosis. Furthermore, we evaluated the immune infiltration profiles of patients with different NKRGS risk levels using immune infiltration analyses. The ssGSEA and xCell algorithms suggested that the high-NKRGS-risk patients demonstrated significantly lower immune cell infiltration and were less likely to yield better anticancer responses from ICI treatments. Meanwhile, the ESTIMATE analysis results further validated that NKRGS risk negatively correlated with immune scores. These findings collectively demonstrated the potential that the NKRGS had in stratifying patients with diverse immune response function. The additional GO and KEGG analyses revealed that the NKRGS highly correlated with the cancer metabolism and immune response pathways, while accumulating evidence suggests that impaired cellular metabolism in the TME contributes to NK cell dysfunction [38,39]. The hypermetabolic state of tumor cells forms a metabolically restrictive TME with high lactate concentrations and low pH values, thereby downregulating NK cell function [40,41,42]. These studies may help explain why NKRGS-related DEGs corelated with the immune response and tumor metabolism pathways in the GO and KEGG analyses.

Several limitations of the present study should be noted. As the construction of the NKRGS was based on transcriptome expression levels, further investigations are warranted to explore the mechanisms of these prognostic genes using in vitro and in vivo experiments. Second, the NKRGS was constructed on NK-cell-related genes; therefore, the predictive power of the NKRGS model might be subjected to TME heterogeneity in different patients and might require further validation.

## 4. Materials and Methods

### 4.1. Data Collection and Patient Selection

The scRNA-seq dataset of immune cells from 5 HCC patients (LIHC_GSE140228_10X) was obtained from the TISH2 database, and the survival profile and expression matrix of HCC dataset as a training cohort were acquired from TCGA database. After excluding patients with fibrolamellar carcinoma (*n* = 3) and combined HCC–intrahepatic cholangiocarcinoma (*n* = 7) and those without survival information (*n* = 6), 355 patients with HCC were included. Furthermore, an additional dataset from the Gene Expression Omnibus (GSE14520, *n* = 242) and our internal bulk RNA-seq cohort (*n* = 116) were included as validation cohorts. Details of the sequencing procedure of our internal bulk RNA-seq cohort were described in a previous work [43]. The baseline characteristics of the three HCC cohorts are shown in Table 1.

### 4.2. Identification of NK-Cell-Related Genes

The downloaded LIHC_GSE140228 scRNA-seq data were analyzed using the seurat package. First, we loaded the scRNA-seq expression matrix to create a seurat object, and the data matrix was filtered using a quality control process. Then, we applied the ScaleData algorithm to normalize the transcriptome data and used the FindVariableFeatures function to screen the top 2000 highly variable genes, thereby applying it to the dimension reduction process. Dimension reduction was carried out using the RunPCA and RunUMAP functions with the top 20 principal components, and cell clusters were also identified using the seurat package. The cell type was confirmed by the top marker genes and acknowledged marker genes of the immune cells. Finally, we integrated the defined cell type information into the established seurat object. The FindAllMarkers function was used to compare the DEGs among the clusters, and we designated genes with expression levels higher than |log (fold change)| > 1 and adjusted *p* values < 0.05 as cluster-related marker genes. NK cell-related genes were extracted from the cluster-related marker genes of the NK cell clusters, and duplicate genes between the clusters were removed.

### 4.3. Development and Validation of the NKRGS

TCGA cohort was used to develop the NKRGS. A univariate Cox analysis was performed on the TCGA cohort to screen prognostic NK-cell-related genes, and genes with *p* values < 0.05 were selected. The LASSO regression [44] was performed to minimize the collinearity effect of the NKRGS. The optimal *λ* was selected to fit the LASSO regression. Then, we performed a multivariate Cox analysis to identify independent prognostic marker genes to develop the NKRGS. The following formula was used to generate the NKRGS risk score:

risk score = Coef(X1) × Exp(X1) + … +Coef(Xn) × Exp(Xn),(2)

where Coef(Xn) and Exp(Xn) refer to the coefficients and expression levels of the marker genes. The patients in TCGA cohort were allocated to either a low- or high-NKRGS-risk group on the basis of their median risk scores, which were estimated using the same formula used for the GSE14520 and internal cohorts for validation. A multivariate Cox analysis was performed to confirm survival significance, while a Kaplan–Meier analysis was used to compare OS between the NKRGS risk groups. A time-dependent ROC curve analysis [45] was applied to assess the prognostic value of the NKRGS, and the AUC represented its predictive performance.

### 4.4. Construction of a NKRGS-Based Nomogram

A nomogram based on the NKRGS was constructed to facilitate clinical utility and assess predictive performance by incorporating other clinicopathological variables. The nomogram was visualized using the rms package and evaluated for survival outcome accuracy based on calibration curves. Furthermore, a time-dependent ROC analysis was performed to evaluate the prognostic value of the NKRGS-based nomogram.

### 4.5. Immune Cell Infiltration Analysis

The heterogeneity of the immunological characteristics between the NKRGS risk groups was investigated using the ssGSEA, and the composition of malignant and immune cells were evaluated using the ESTIMATE analysis. The correlations between the immune cells and the NKRGS were assessed using the xCell algorithm [46].

### 4.6. Functional Enrichment Analysis

The DEGs between the NKRGS risk groups were identified with the limma package, with the same thresholds applied in filtering NK-cell-related genes. These NKRGS-related DEGs were selected for the KEGG and GO analyses, which were aimed at identifying NKRGS-related biological functions or pathways.

### 4.7. Statistical Analysis

All analytical procedures were performed using the R software version 4.1.1 (The R Foundation, Vienna, Austria), and statistical significance was defined as a two-tailed *p* value < 0.05.

## 5. Conclusions

We established a novel NK-related gene prognostic model that can stratify the prognosis of patients with HCC, and revealed the immunosuppressive status of patients with high NKRGS risk. Higher KLRB1 and DUSP10 expression levels correlated with the favorable survival of the patients with HCC.

## Figures and Tables

**Table 1:** Baseline characteristics of the patients with HCC in the three cohorts.

Variable	TCGA Cohort *n* = 355	Internal Cohort *n* = 116	GSE14520 Cohort *n* = 242
Age, years			
≤60	169	101	196
>60	186	25	46
Sex			
Male	240	101	211
Female	115	15	31
Grade			
I–II	222	59	NA
III–IV	128	57	NA
Unknown	5	0	NA
Alpha-fetoprotein, ng/mL			
≤400	207	45	NA
>400	62	71	NA
Unknown	86	0	NA
TNM stage			
I–II	246	NA	174
III–IV	85	NA	51
Unknown	24	NA	17
BCLC stage			
0–A	NA	58	172
B	NA	32	24
C	NA	26	29
Unknown	NA	0	17
Vascular invasion			
Micro	86	50	NA
Macro	16	23	NA
None	199	43	NA
Unknown	54	0	NA
HBV/HCV infection			
Yes	NA	99	218
No	NA	17	6
Unknown	NA	0	18

BCLC, *Barcelona Clinic Liver Cancer*; HBV/HCV, hepatitis B/C virus; NA, not available.

## Data Availability

TCGA-LIHC (https://portal.gdc.cancer.gov, (accessed on 17 February 2023)), LIHC_GSE140228 (http://tisch.comp-genomics.org/home/, (accessed on 2 March 2023)), and GSE14520 (https://www.ncbi.nlm.nih.gov/geo/, (accessed on 2 March 2023)) datasets are available online. The RNA sequencing data of our internal cohort are available from the corresponding author upon reasonable request.

## Supplementary Materials

The supporting information can be downloaded at: https://www.mdpi.com/article/10.3390/ijms24119587/s1.
