# Supplementary figures and images for: The Novel-Natural-Killer-Cell-Related Gene Signature Predicts the Prognosis and Immune Status of Patients with Hepatocellular Carcinoma
Source: Int J Mol Sci. 2023 May 31;24(11):9587. doi: 10.3390/ijms24119587 (PMC10253630; doi:10.3390/ijms24119587)

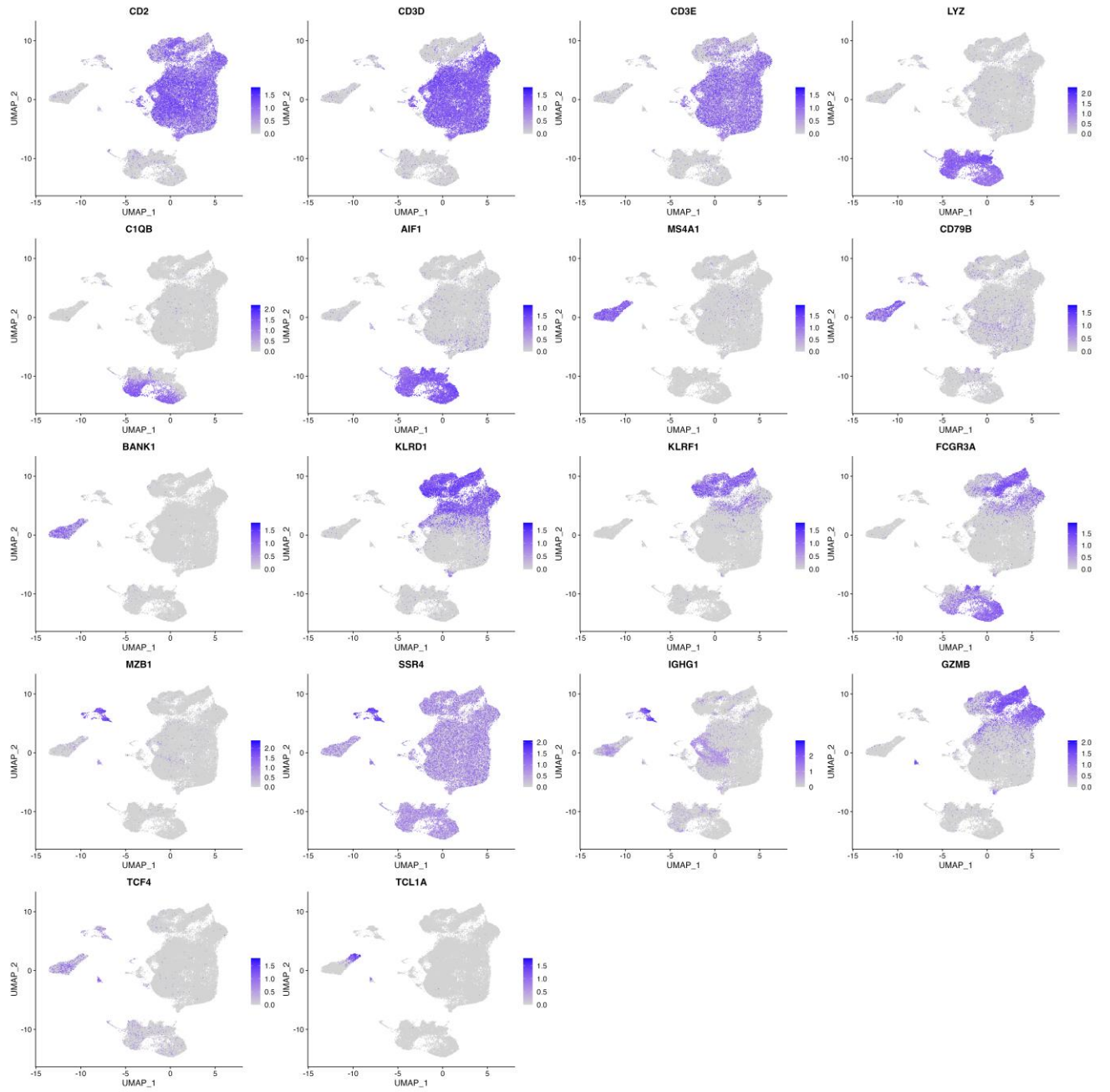

**Figure S1.** Expression profiles of marker genes for cell type definition.

Supplement: Supplementary file 1 [file ijms-24-09587-s001.zip › ijms-2362331-supplementary.pdf]
